# A novel method to derive a human safety limit for PFOA by gene expression profiling and modelling

**DOI:** 10.3389/ftox.2024.1368320

**Published:** 2024-03-21

**Authors:** Arthur de Carvalho e Silva, George D. Loizou, Kevin McNally, Olivia Osborne, Claire Potter, David Gott, John K. Colbourne, Mark R. Viant

**Affiliations:** ^1^ School of Biosciences, University of Birmingham, Birmingham, United Kingdom; ^2^ Centre for Environmental Research and Justice (CERJ), University of Birmingham, Birmingham, United Kingdom; ^3^ Health and Safety Executive, Buxton, United Kingdom; ^4^ Science Evidence and Research Division, Food Standards Agency, London, United Kingdom

**Keywords:** PFOA, PBK, *in silico*, Bayesian, Markov chain Monte Carlo, reverse dosimetry, omics, NAMs

## Abstract

Perfluorooctanoic acid (PFOA) is a persistent environmental contaminant that can accumulate in the human body due to its long half-life. This substance has been associated with liver, pancreatic, testicular and breast cancers, liver steatosis and endocrine disruption. PFOA is a member of a large group of substances also known as “forever chemicals” and the vast majority of substances of this group lack toxicological data that would enable their effective risk assessment in terms of human health hazards. This study aimed to derive a health-based guidance value for PFOA intake (ng/kg BW/day) from *in vitro* transcriptomics data. To this end, we developed an *in silico* workflow comprising five components: (i) sourcing *in vitro* hepatic transcriptomics concentration-response data; (ii) deriving molecular points of departure using BMDExpress3 and performing pathway analysis using gene set enrichment analysis (GSEA) to identify the most sensitive molecular pathways to PFOA exposure; (iii) estimating freely-dissolved PFOA concentrations *in vitro* using a mass balance model; (iv) estimating *in vivo* doses by reverse dosimetry using a PBK model for PFOA as part of a quantitative *in vitro* to *in vivo* extrapolation (QIVIVE) algorithm; and (v) calculating a tolerable daily intake (TDI) for PFOA. Fourteen percent of interrogated genes exhibited *in vitro* concentration-response relationships. GSEA pathway enrichment analysis revealed that “fatty acid metabolism” was the most sensitive pathway to PFOA exposure. *In vitro* free PFOA concentrations were calculated to be 2.9% of the nominal applied concentrations, and these free concentrations were input into the QIVIVE workflow. Exposure doses for a virtual population of 3,000 individuals were estimated, from which a TDI of 0.15 ng/kg BW/day for PFOA was calculated using the benchmark dose modelling software, PROAST. This TDI is comparable to previously published values of 1.16, 0.69, and 0.86 ng/kg BW/day by the European Food Safety Authority. In conclusion, this study demonstrates the combined utility of an “omics”-derived molecular point of departure and *in silico* QIVIVE workflow for setting health-based guidance values in anticipation of the acceptance of *in vitro* concentration-response molecular measurements in chemical risk assessment.

## 1 Introduction

Perfluorooctanoic acid (PFOA) is a fully fluorinated substance largely used in the manufacture of consumer products including food wrappers, non-stick cookware, and coatings (Bartell et al., 2010). PFOA is also known as a “forever chemical” due to its persistence both in the environment as it breaks down very slowly and potential to bioaccumulate in the human body (Rayne and Forest, 2009; Bartell et al., 2010; Shin et al., 2011). At a mechanistic level, PFOA has been implicated in increasing gene expression involved in cholesterol biosynthesis and metabolism (Fletcher et al., 2013; Louisse et al., 2016). Additionally, recent epidemiological studies identified critical effects on the immune system (Bampidis et al., 2020), which may result from a dysregulated cytokine/chemokine response and impaired neutralising antibody response (Lee et al., 2017). Current EU regulation (Article 25, EC 2020/2184) provides that member states shall take the necessary measures to ensure safe levels of a sum of per- and polyfluoroalkyl substances in water intended for human consumption by 2026. Given these effects, a thorough risk assessment of PFOA, including the derivation of an accurate health-based guidance value remains an important need.

Momentum for a paradigm shift in chemical risk assessment is growing (Hartung and Tsatsakis, 2021). Reduction in animal testing and its replacement with non-animal testing procedures received considerable impetus following the enforcement of the European Union (EU) Cosmetics Regulation (EC 1223/2009) in 2013. This directive imposed a full marketing ban in Europe for cosmetic products and ingredients tested on animals anywhere in the world. Other directives such as EC 2010/63/EU (amended by Article 6 of Regulation 2019/2010) and REACH (EC 1907/2006) also strengthen political pressure for progress towards replacing animal testing in science and the use of alternative test methods for hazard assessment of chemicals.

Alternative test methods that seek to minimise animal testing, known as New Approach Methodologies (NAMs), include *in silico* methods and their integration with *in vitro* bioassays that use human cells for determining a molecular point of departure (PoD). In the context of New Generation Risk Assessment (NGRA) (Johnson et al., 2022; Barutcu et al., 2023), the application of “omics” technologies to derive PoDs has been growing rapidly (Farmahin et al., 2017; Shah et al., 2021; Alcaraz et al., 2022; Beal et al., 2022; Jin et al., 2022; Kuo et al., 2022; Gou et al., 2023; Malinowska et al., 2023; Min et al., 2023; Ramirez-Hincapie et al., 2023; Reardon et al., 2023; Song et al., 2023). PoDs are used as the basis for the derivation of safe levels of human exposure known as Health Based Guidance Values (HBGV) (Ingenbleek L et al., 2021).

The benchmark dose (BMD) approach fits dose–response models to a complete dose–response dataset to identify the dose that corresponds to the lowest biologically relevant change in the response (i.e., benchmark response or BMR). The BMD is reported with a credible interval, e.g., the lower and upper confidence limits of the benchmark dose (BMDL and BMDU) for a selected observed level of effect (More et al., 2022).

The BMD is increasingly preferred by regulatory agencies, but its use is often limited by test design, which in turn are limited by current OECD guidelines for *in vivo* assays (Bokkers and Slob, 2005; Bokkers and Slob, 2007; European Food Safety, 2009; Hardy et al., 2017).

*In vitro* concentration-response data must be converted to *in vivo* dose-responses for use in human safety testing of chemicals. This process is known as quantitative *in vitro* to *in vivo* extrapolation (QIVIVE) (Yoon et al., 2012; Bale et al., 2014; Yoon et al., 2015). QIVIVE increasingly involves the application of physiologically based kinetic (PBK) modelling. In most previous QIVIVE studies, all PBK parameters other than input dose or exposure were fixed at central values. The discrepancy between a target *in vivo* dose, predicted by the PBK model, and a given *in vitro* concentration was minimised by an optimisation routine. The concentration corresponding to the target *in vitro* concentration was considered a surrogate for the *in vivo* dose. These studies, however, did not account for structural uncertainty in the PBK model, nor parameter value uncertainty (Louisse et al., 2010; Louisse et al., 2012; Strikwold et al., 2013; Louisse et al., 2016; Boonpawa et al., 2017a; Boonpawa et al., 2017b; Li et al., 2017; Punt et al., 2017; Strikwold et al., 2017; Adam et al., 2019; Zhao et al., 2019; Shi et al., 2020; Zhang et al., 2020). It is also well known that the amount of biological mechanistic detail described in a PBK model can have a bearing on model output (Rowland et al., 2017; McNally et al., 2018; Loizou et al., 2021).

Another limitation of earlier QIVIVE studies is the use of applied or nominal *in vitro* concentrations. This means that no consideration is made of the fate and distribution of the chemical in the *in vitro* test system. This could be a significant omission because the concentrations of chemical that are bioavailable to interact with the cells could be substantially lower than the nominal concentration (Tanneberger et al., 2010; Armitage et al., 2014; Groothuis et al., 2015; Kramer et al., 2015; Armitage et al., 2021; Proença et al., 2021). Chemicals that are highly lipophilic are particularly prone to interacting with constituents of the reaction medium and reaction vessel geometry and composition (Armitage et al., 2014; Proença et al., 2019; Armitage et al., 2021; Proença et al., 2021). Currently, there are several *in silico* tools that calculate the proportion of the nominal concentration that is free and presumably active (e.g., the *in vitro* mass balance Armitage model), and therefore available in the reaction medium to be taken up by cells (Tanneberger et al., 2010; Armitage et al., 2014; Comenges et al., 2017; Fischer et al., 2017; Fisher et al., 2019; Armitage et al., 2021). The Armitage model can estimate: (i) chemical fate and transport; (ii) cell partitioning; (iii) sorption to the reaction vessel; and (iv) considers the experimental set up (size of well-plate). Ultimately, it can simulate the active, free concentration of a chemical in the cell medium, which can be assumed to be equal to the organ or tissue efferent concentration, that is, the concentration exiting the organ or tissue and entering the venous blood compartment described in the PBK model. The Armitage model can also be used on its own to design and interpret *in vitro* experiments, and in combination with PBK models to perform *in vitro* to *in vivo* extrapolation (Comenges et al., 2017; Bell et al., 2018; Armitage et al., 2021). It is important to mention that the mass balance model has its own limitations and although it accounts for cellular uptake and excretion, it does not consider metabolic activity or the activity of specific cell transporters.

Here, we calculate a HBGV from the *in vitro* benchmark concentration modelling of previously published gene expression changes in human primary liver cell spheroids mediated by PFOA (Rowan-Carroll et al., 2021). The *in vitro* transcriptomics data were translated to *in vivo* dose-responses with a PBK model for PFOA (Worley et al., 2017; Loizou et al., 2021). A TDI (ng/kg BW/day) was calculated from the *in vivo* dose-responses and compared to the TDI values recommended by the European Food Safety Authority (EFSA) in the Scientific Opinion on the Risk to human health related to the presence of perfluoroalkyl substances in food for effects on the immune system (Bampidis et al., 2020) and previously for increases in serum cholesterol (Knutsen et al., 2018). Our work was achieved using a QIVIVE workflow described previously for ethylene glycol monoethyl ether (EGME) (McNally et al., 2018), PFOA (Loizou et al., 2021) and bisphenol A (Loizou et al., 2022) that accounts for PBK model structure uncertainty, parameter value uncertainty, and the calculation of free concentration of chemical *in vitro* by applying a rigorous statistical (probabilistic) framework to accommodate uncertainties. Overall, the purpose of this study, however, was not to propose an updated animal-free risk assessment for PFOA, since we recognise that much work is still needed to demonstrate *in vitro* to *in vivo* concordance for systemic, chronic exposures to environmental xenobiotics. Such a demonstration is beyond the scope of this study. Instead, this study serves to further demonstrate and build confidence in the utility of “omics”-derived molecular PoD measurements in combination with the multi-step *in silico* QIVIVE workflow in anticipation of the acceptance of *in vitro* concentration-response data in chemical risk assessment.

## 2 Materials and methods

**FIGURE 1:**
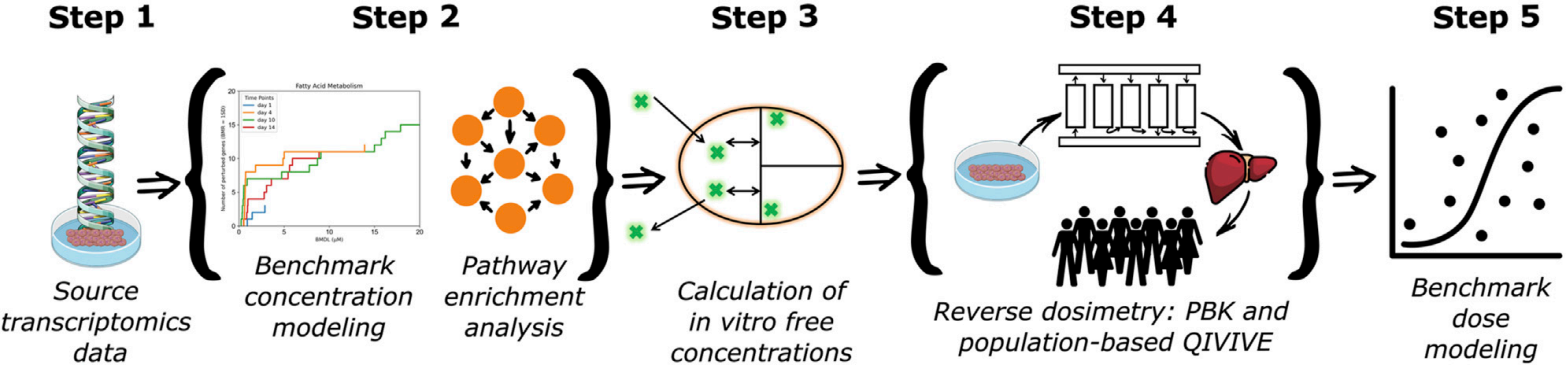
Workflow for the integration of *in vitro* and *in silico* new approach methodologies to derive health-based guidance values for the risk assessment of a chemical.

The step-by-step workflow used in this study is depicted in Figure 1. Each of the following subsections are mapped according to the five steps in our workflow.

### 2.1 Step 1: sourcing transcriptomics data

Gene expression changes mediated by varying concentrations of PFOA were investigated previously by analysing the high-throughput *in vitro* transcriptomic data of (Rowan-Carroll et al., 2021). Briefly, the authors measured transcriptomic responses for 2977 genes in liver spheroids cultured under standard conditions and treated for 1, 4, 10, or 14 days to PFOA, or DMSO (the control condition) diluted in culture medium. Final nominal exposure concentrations were 0.02, 0.1, 0.2, 1, 2, 10, 20, 50 and 100 µM in 96-well plates. The transcriptomic data from the final concentration of 100 µM was excluded from our study, due to the reported cytotoxicity (Rowan-Carroll et al., 2021). We downloaded the transcriptomic data from https://www.ncbi.nlm.nih.gov/geo/query/acc.cgi?acc=GSE144775 which were analysed according to the steps described by the authors, integrating the R package DESeq2 (Love et al., 2017) into the KNIME Analytics Platform v. 4.6.4. (Berthold et al., 2009). The intensity data were then imported to BMDExpress3 (Wheeler et al., 2023).

### 2.2 Step 2: benchmark concentration modelling and pathway enrichment analysis to identify the most sensitive pathways

In our study, we decided to visualise and rank the most sensitive genes by generating benchmark dose models for each gene in the gene set. BMDExpress3 software was used to generate benchmark dose models for each gene in the transcriptomics dataset. Hill, power, exponential 3 and exponential 5 models were fitted using the ToxicR Model Averaging function using a BMR factor of 1 standard deviation. To filter only genes exhibiting reasonable dose-response relationships, the following criteria were used: (i) Best fit *p*-value >0.001, (ii) BMDU/BMDL ≤40, (iii) data producing benchmark concentration values lower than or equal to the highest nominal exposure concentration (50 µM).

The BMDExpress3 results for the whole gene set containing 2977 genes were ranked from the lowest to highest BMDL values. Next, Gene Set Enrichment Analysis (GSEA) v. 4.3.2 (Subramanian et al., 2005) was used to cross-link the entrez gene numbers for each gene in the gene set with the MolSigDB hallmark gene set collection (Liberzon et al., 2011), and to enrich the most significant pathways against the pre-ranked genes using the “GSEAPreranked” module. All duplicated probes were collapsed and their BMDL values were averaged. To ensure reproducibility, the parameter “seed for permutation” was set to 149 instead of “timestamp”. The default values for the other parameters (e.g., number of permutations, maximum and minimum sizes, collapsing mode for probe sets, and normalization mode) were applied. Pathways were ordered by FDR q-values (Benjamini and Hochberg, 1995) and only pathways with q < 0.1 were deemed to be significant. The most sensitive gene of each statistically significant pathway, i.e., presenting the lowest BMDL value, was selected for the next step of the workflow.

### 2.3 Calculation of *in vitro* free concentrations of PFOA

The updated version of the *in vitro* mass balance model (Armitage et al., 2021) was used to estimate the *in vitro* freely dissolved PFOA concentrations. This is an approach to simulate the distribution of a chemical of interest when tested *in vitro*. Briefly, the whole test system is described as compartments, mainly composed of the headspace of the well, the plastic of the microplate, the medium lipids and proteins, and the cells. At the end of the process, the *in vitro* mass balance model estimates the fate of the chemical and provides the percentage of the chemical that is freely dissolved and the percentages interacting with each compartment. The mass balance model was implemented as a macro in Microsoft Excel^®^ and requires the following physical-chemical properties to be provided by the user (see Supplementary Table S1, Supplementary Material S2): molecular weight (g/mol), melting point (°C), pKa, octanol-water partition ratio of the neutral form (log KOW,N), air-water partition ratio of neutral form (log KAW,N), and water solubility (mg/L). Due to the lack of parameters for liver spheroids in the current version of the mass balance model, the test system parameters (e.g., FBS volume fraction, microplate size) were also provided using the details of a ToxCast assay (AEID46) with HepG2 cells exposed to PFOA. All nominal concentrations were input in units of µM. All these physical-chemical property values for PFOA were obtained from the US EPA EPI Suite (US EPA, 2012).

### 2.4 Steps 4 and 5: reverse dosimetry—PBK model and QIVIVE algorithm—and benchmark dose modelling to derive a health-based guidance value

The generic PBK model for PFOA described previously (Loizou et al., 2021), written in the GNU MCSim syntax (version 6.1.0)^1^ was executed under Windows 10 Pro using RStudio (RStudio Team, 2016). The files for executing MCSim under windows, with tools and instructions for installation are available from Github^2^. Parameterisation, sensitivity and uncertainty analysis, and calibration were described previously (Loizou et al., 2021).

This statistical framework also incorporates global sensitivity analysis (GSA) of PBK models, Approximate Bayesian Computation (ABC), and a Markov Chain Monte Carlo (MCMC) simulation to convert *in vitro* concentration-response data to *in vivo* dose-response data (McNally and Loizou, 2015; McNally et al., 2018; Loizou et al., 2021; Loizou et al., 2022). Understanding and quantifying these uncertainties in each step of a chemical risk assessment with NAMs is important for building confidence in this approach (Berggren et al., 2017).

The ABC statistical framework was used in this work to perform a reverse dosimetry, i.e., obtain the equivalent *in vivo* doses from the *in vitro* concentrations that the liver spheroids were exposed to in the study published by (Rowan-Carroll et al., 2021). This statistical framework was also used to generate a virtual population of 3,000 individuals, to calculate the concentrations of PFOA in the drinking water, and to calculate the intake (ng/kg BW/day) of PFOA for the virtual population.

The following formula was used to calculate the intake:

$$Intake=\frac{{\left[PFOA\right]}_{DW}\times {DW}_{total}}{BW}$$

Where [PFOA]_DW_ is the concentration of PFOA in drinking water in µg/L, DW_total_ is the total daily drinking water consumption in L, and BW represents the body weight in kg.

The [PFOA]DW was obtained as means of the 3,000 virtual individuals for each concentration used by Rowan-Carrol et al. (2021) and used as input to derive a HBGV for PFOA using EFSA’s PROAST web application.

EFSA’s open analytics tool hosts PROAST, which was developed by the Netherlands National Institute for Public Health and the Environment (RIVM) (https://r4eu.efsa.europa.eu/app/bmd) (Slob 2018), was used with its default configurations to derive the TDI for PFOA. The number of bootstrap pseudo-replication of the data to calculate model-averaged BMD confidence intervals was 200, and the maximum difference in Akaike information criterion (AIC) was 2. The critical effect size, or benchmark response, was set to 5% and the confidence level to estimate the BMD confidence intervals was defined as 90%.

The use of a population-based PBPK model for PFOA published by (Loizou et al., 2021) accounts for human toxicokinetic variability, therefore an uncertainty factor for this component was not applied. To derive the final benchmark dose, the result obtained from PROAST was divided by the uncertainty factor (UF) of 3.16, to account for human toxicodynamic variability.

## 3 Results

### 3.1 Benchmark concentration modelling and pathway enrichment analysis to identify the most sensitive pathways

From the statistical analysis of the transcriptomics data, day 10 of PFOA exposure exhibited the largest number of differentially expressed genes in the liver spheroids (see Supplementary Table S2). Therefore, this was our starting point to fit BMD models and derive a PoD for each of the 2977 measured genes. After fitting the concentration-response models and performing model averaging, using BMDExpress3, 429 genes out of the 2977 measured gene set (14%) passed the defined filtering criteria and were flagged as responsive.

For the purpose of enriching as many putative response pathways as possible, we chose to also retain in our analysis the statistically non-responsive genes. Therefore, the list containing all 2977 genes and their respective BMDL values was exported, and the genes were ranked from the most to the least sensitive. After importing the list into GSEA and running the “GSEAPreranked” module, the duplicated probes were collapsed and averaged, resulting in a final list of 2591 unique genes. The “GSEAPreranked” module does not consider any phenotype information. However, GSEA still generates two separated outputs. The first shows results for gene sets that have a positive enrichment score or enrichment at the top of the ranked list. The second shows results for gene sets that have a negative enrichment score or enrichment at the bottom of the ranked list. Both runs cross-check the gene names against the hallmark gene collection of MolSigDB 3.0 and generate a list of genes within the leading edge that were significant at a false discovery rate (FDR) lower than 25%. The list of statistically relevant pathways came from the first output. Table 1 shows the four pathways with q < 0.1, and these were retained for further analysis.

**TABLE 1:** Statistically significant pathways enriched after executing the GSEA module using as input our pre-ranked gene list. False discovery rate (FDR) is expressed as q-value and pathways with q-value <0.1 were considered statistically significant. Benchmark dose lower (BMDL) limit values are expressed in μM for both nominal and freely dissolved concentrations.

Pathway	Total number of genes	Number of genes (leading edge)	FDR q-value	Top 5 most sensitive genes	BMDL (nominal concentrations µM)	BMDL (estimated free concentrations µM)
MYC TARGETS V2	16	13	0.03	CDK4	10.37	0.30
				HSPD1	12.02	0.35
				WDR43	13.69	0.40
				HSPE1	13.96	0.40
				EXOSC5	14.43	0.42
FATTY ACID METABOLISM	60	55	0.037	ECH1	0.25	0.01
				ACOX1	0.37	0.01
				ACSL1	0.39	0.01
				FABP1	0.47	0.01
				CYP4A22	0.47	0.01
MYC TARGETS V1	82	67	0.051	C1QBP	10.15	0.29
				CDK4	10.37	0.30
				HSPD1	12.02	0.35
				MCM7	12.32	0.36
				TOMM70	12.95	0.38
UNFOLDED PROTEIN RESPONSE	48	38	0.09	TUBB2A	6.79	0.20
				EEF2	10.01	0.29
				IFIT1	12.90	0.37
				VEGFA	14.04	0.41
				PSAT1	14.14	0.41

The list of statistically relevant pathways was compiled, and their most sensitive genes were identified. The fatty acid metabolism pathway contained the most sensitive genes. While Table 1 lists only the top 5 most sensitive genes, it is important to note that the BMDL values are <1 µM for the first 9 genes in this pathway, which suggests that at least 9 genes of the fatty acid metabolism pathway are perturbed at concentrations below 1 μM. ECH1 was the most sensitive gene of that pathway, and it was also the most sensitive gene in two other pathways—“cholesterol homeostasis” and “oxidative phosphorylation” (see Supplementary Material S1).

**FIGURE 2:**
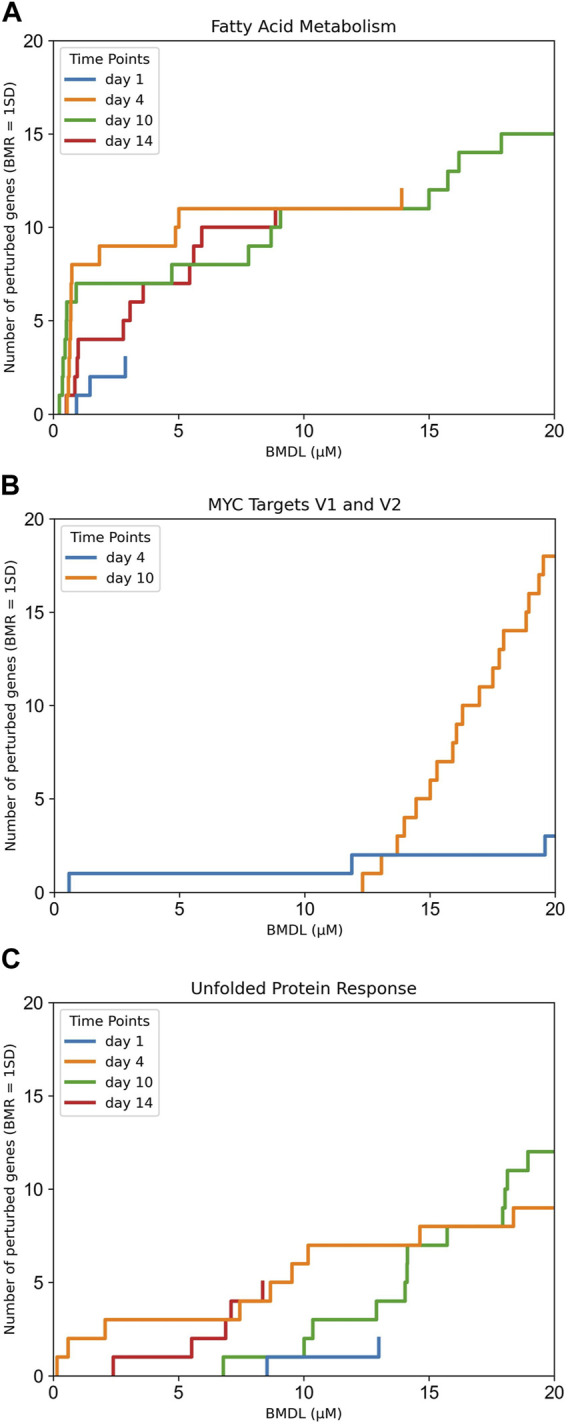
Gene accumulation plots for the enriched pathways derived using BMDExpress3 concentration-response modelling of transcriptomics data derived from the exposures of human primary liver cell spheroids to PFOA. **(A)** Fatty acid metabolism pathway, which was identified as the most sensitive pathway using gene set enrichment analysis; **(B)** MYC Targets V1 and V2; and **(C)** unfolded protein response. Colours indicate the duration of exposure to PFOA, highlighting the consistent effects on fatty acid metabolism after 4, 10 or 14 days of exposure.

There were two cancer-related pathways (pro-proliferative and anti-apoptotic) MYC Targets V1 and V2 that share most of their gene sets, which are perturbed at nominal concentrations between ca. 10–15 µM. This same concentration range also seems to activate the unfolded protein response pathway, which is related to endoplasmic reticulum (ER) homeostasis (Harding et al., 2000; Harding et al., 2003; Zhao and Ackerman, 2006). Figure 2 shows a comparison of the gene accumulation plots for each pathway.

The fatty acid metabolism pathway, a PPAR-related pathway, as shown in Table 1, is described as part of the human hallmark gene set collection, comprising 158 genes (Liberzon et al., 2011; Liberzon, 2014; Liberzon et al., 2015), yet only 60 genes in this pathway were investigated in the transcriptomics data, and the “GSEAPreranked” module results demonstrated that 55 of these genes contributed to the core enrichment of this pathway. The mean and median BMDL for the 60 genes in this pathway were respectively 23.05 and 23.17 µM, while the maximum BMDL was 41.18 µM, for the gene GSTZ1. The BMDL value for PPARα was 17.33 µM.

### 3.2 Estimation of *in vitro* free concentrations

The *in vitro* mass balance model estimated the freely dissolved concentration of PFOA at 2.9% of the nominal applied concentration. Therefore, we have multiplied each nominal concentration by a factor of 0.029 to obtain the free PFOA concentrations (see Table 1) and proceed with the QIVIVE step of our workflow.

### 3.3 Steps 4 and 5: reverse dosimetry—PBK model and QIVIVE algorithm

The *in vivo* dose responses obtained after running the QIVIVE workflow were used to estimate a BMDL for the most sensitive gene in the fatty acid metabolism pathway, ECH1. The BMDL derived is shown in Table 2 along with other values previously derived for PFOA and published by EFSA and our group.

**TABLE 2:** Comparison between tolerable daily intake (TDI) values derived in this study and previously published health-based guidance values.

PFAS	Critical effects	TDI (ng/kg bw per day)	References
PFOA, PFOS, PFNA, PFHxS	Immune system	1.16	Bampidis et al. (2020)
PFOA	Serum cholesterol	0.86	Knutsen et al. (2018)
PFOA	Antibody titre	0.69	
PFOA	Gene expression data—ECH1	0.15	Current study
	ToxCast (Serum cholesterol)	0.19	Loizou et al. (2021)
	ToxCast (Antibody titre)	1.55	Loizou et al. (2021)

The BMDL of 0.15 ng/kg BW/day was approximately 5-fold lower than the 0.86 ng/kg BW/day for serum cholesterol and 4.6-fold lower than the 0.69 ng/kg BW/day for antibody titre derived by EFSA’s scientific panel for food contaminants in 2018 (Knutsen et al., 2018). It is also 7-fold lower than the value recently published by EFSA for the sum of four different PFAS (PFOA, PFOS, PFNA, and PFHxS) as food contaminants (Bampidis et al., 2020). Further, the value derived in this study is similar to the BMDL value derived for serum cholesterol and ten-fold lower than the value derived for antibody titre by (Loizou et al., 2021).

## 4 Discussion

In the foreseeable future, risk assessments are increasingly likely to incorporate animal-free testing methods and gain acceptance. There are extensive and ongoing efforts worldwide to increase the uptake of NAMs, including to gain their acceptance by regulatory agencies. Next-Generation Risk Assessment (NGRA)-aligned frameworks embrace the notion of “protecting not predicting”, which has the goal of protecting humans from harmful exposure levels to a plethora of substances found in the environment, food, drinking water, and other sources. This is where this piece of work is positioned—aligned with NGRA principles, considering the uncertainties of each method integrated into the proposed *in silico* workflow, and demonstrating the utility of well-documented NAMs within a case study.

Our work builds on the gene expression data generated by (Rowan-Carroll et al., 2021) upon exposure of liver spheroids to different concentrations of four PFAS, including PFOA, over a time course of 14 days. Our study also relied on a probabilistic approach that combines a calibrated PBK model (Loizou et al., 2021) and a population based QIVIVE algorithm for deriving exposure doses. There are several advantages regarding exposure or dose reconstruction provided by this probabilistic approach. First, by defining informative prior distributions around parameters, a deterministic model converts to a population model, which can account for inter-individual variability. Second, the probabilistic approach is appropriate for systems where tissue dose is not necessarily linearly related to external exposure. For instance, there is evidence demonstrating that interindividual variability of blood protein concentrations can affect the relationship between external exposure and internal doses of PFAS due to their particular toxicokinetic behaviour (Fischer et al., 2024). Because per- and polyfluoroalkyl substances tend to bind to serum proteins and nuclear receptors, their half-lives also tend to be increased; on the other hand, if a disease lowers the quantity of serum proteins, there will be more unbound PFAS circulating, hence a higher susceptibility to their adverse effects is also expected (Zaccherini and Bernardi, 2019; Nielsen et al., 2024; Rosato et al., 2024). Finally, this combination can extract population variability and multiple routes of exposure information integrated within pharmacokinetic data (McNally et al., 2012; McNally et al., 2018).

We propose a solution to the oftentimes arbitrary choice of a biologically relevant molecular PoD made from the observed perturbations of single genes by first determining the most sensitive pathways to the chemical exposure via a gene set enrichment analysis of functionally linked gene responses. To fulfil this approach, we derived molecular PoDs from the publicly available transcriptomics data using BMDExpress3, which in turn allowed for ranking genes according to their BMDL values. The question we sought to answer was straightforward; our aim was to demonstrate the utility of an *in silico* workflow designed to extrapolate *in vitro* concentration-response data related to the responses of the most sensitive and statistically relevant pathways activated by exposure to low, environmentally-relevant concentrations of PFOA to *in vivo* doses. The pathway enrichment analysis highlighted “fatty acid metabolism”, “MYC targets V1 and V2”, and “unfolded protein response” pathways as the most sensitive to PFOA exposure. ECH1 was the most sensitive gene among the 60 genes in the fatty acid metabolism pathway. The BMDL value for ECH1 (0.25 µM) was 70-fold lower than the BMDL for PPARα (17.33 µM), which is consistent with studies demonstrating the relation between PFOA exposure and activation of PPAR-related pathways (Kaiser et al., 2022).

PPAR-related pathways play an important role in lipid metabolism in humans; PPAR receptors family is composed by three subtypes—α, δ, and γ—and differ in their spectrum of activities. PPAR α is mainly expressed in the liver, but can also be found in the heart, bones, and muscles (Kamata et al., 2023). PPARα activation has been described by Yang et al. (2023) as an important molecular initiating event in PFOA/PFOS induced dysregulation of hepatic lipid metabolism both in humans and rodents; besides, the authors also demonstrated that hepatic ACOX1 in the peroxisomal β-oxidation pathway is also a critical molecular target associated with PFOA/PFOS dysregulated lipid metabolism and resultant hepatotoxicity. In this work, we identified ACOX1 as the second most sensitive gene, with a BMDL of 0.37 µM, and the gene set enrichment analysis recognised it as part of the fatty acid metabolism pathway.

BMDL concentrations lower than 1 µM activated responses in at least 9 genes in the fatty acid metabolism pathway, suggesting the onset of molecular events that could eventually lead to downstream phenotypic outcomes. Such low concentrations derived from gene expression data are anticipated to translate to conservative TDIs using our workflow to integrate transcriptomics concentration-response data with a calibrated PBK model. These results are also useful for the study of molecular key events of relevant adverse outcome pathways and for the question of how many genes need to be initially perturbed for the test system to be considered perturbed.

The TDI derived using our computational workflow was in the same range and in general slightly more conservative than the values derived by EFSA. Loizou and colleagues (2021) applied the same workflow, using *in vitro* concentration-response data from the ToxCast/Tox21 database. That study focused on concentration-response data related to estrogen, pregnane X and thyroid receptor binding, associated with breast cancer, altered serum cholesterol levels, thyroid disease/decreased antibody titre. As shown in Table 2, the TDI derived using transcriptomics data, which interrogates a broader range of molecular responses, was more conservative than those values derived using cell-based high-throughput screening *in vitro* data.

An aim of this study was to demonstrate the utility of a workflow designed to quantify uncertainty within the different stages of data analysis and processing and to account for human inter-individual variability. The health-based guidance value determined in this study is not proposed for the future regulation of PFOA. Further work is required to provide the confidence in the use of *in vitro* to *in vivo* NAMs required by regulatory agencies. Furthermore, this study has a number of limitations. Firstly, the absence of parameters for liver spheroids in the current version of the mass balance model used in this work. Considering that the liver spheroids were composed of primary human hepatocytes and Kupfer cells, we have addressed this limitation by using the parameters for HepG2 cells according to standard parameters of an *in vitro* ToxCast assay for PFOA. This limitation is linked to the uncertainty around the freely dissolved concentrations of PFOA, as our approach considered a common cell culture and not a spheroid-based biological test system.

Another important limitation to be discussed is how different mathematical (*in silico*) models address the estimation of the freely dissolved fraction of a chemical within an *in vitro* test system. Different methods with different parameters have been proposed and to determine the most adequate method to estimate the mass balance of the different chemical classes is anything but trivial (Proença et al., 2021). Moreover, determining the applicability domain of such models is also challenging. In our study, we have tested both the updated version of the *in vitro* mass balance model (Armitage et al., 2021) and the virtual cell-based assay (VCBA) model (Proença et al., 2019). We decided to proceed with the updated version of Armitage’s model (Armitage et al., 2021) as it had the capability to simulate a ToxCast assay with larger microplate settings and handle ionizable organic compounds such as PFOA. It is noteworthy that the correction of the nominal dose to derive an available fraction is an important step of the workflow. We have reviewed some of the available models, their data requirements, and limitations in the introduction, but a broader review can be found elsewhere (Proença et al., 2021). As with all models there is uncertainty associated with predictions, and different models may be better for certain classes of chemicals. A particular model may have both uncertainty and a bias in predictions of the freely dissolved fraction for certain chemical classes (Heringa et al., 2004; Teeguarden and Barton, 2004; Treijtel et al., 2004; Treijtel et al., 2005; Madureira et al., 2014; Kwon et al., 2020) or may be more general (Gulden and Seibert, 2003; Armitage et al., 2014; Stadnicka-Michalak et al., 2014; Comenges et al., 2017; Fischer et al., 2017; Fisher et al., 2019). In this work there has been no explicit consideration of uncertainty and a potential bias in the calculations for the free fraction. However, we note that model uncertainty, arising from a multitude of (unspecified) sources, is explicitly considered in the QIVIVE through the ABC algorithm, therefore we consider this uncertainty to be already addressed within the workflow. Any uncertainty arising from the correction of the nominal dose is in any case trivially small relative to the case of an uncorrected nominal concentration used within calculations.

In the present work, despite the lack of specific parameters for liver spheroids in the current version of Armitage’s model (Armitage et al., 2021), the freely dissolved concentrations of PFOA was estimated to be 2.9% of the nominal concentrations. Previously (Loizou et al., 2021), tried to predict the freely dissolved concentrations of PFOA as 2.3% of the nominal concentrations based on its LogP. It was not based on any specific model, but solely relying on the estimations for benzo [a]pyrene (which has a similar LogP to PFOA) calculated and published by (Proença et al., 2019).

Another important limitation is that our *in vivo* benchmark calculation accounted for the data of just one exposure time point of the original study (day 10), with a visual comparison with the other time points. The transcriptomics data is functional; however, it is a snapshot of the exposure to a chemical at that time point. As depicted in Figure 2, the number of perturbed genes in the fatty acid metabolism pathway increases and reaches its maximum at day 10, and decreases on day 14, as the gene expression pattern returns towards baseline.

Kaiser et al. (2022) mapped PFOA to AOPs using the AOP-helpFinder tool (http://aop-helpfinder.u-paris-sciences.fr/index.php) (Kaiser et al., 2022). The concept of an AOP is to relate the knowledge about a molecular initiating event (MIE) with an adverse outcome (AO) through several key events (KE), connected by key events relationships, occurring at different biological levels (Ankley et al., 2010). Their study highlighted the relation between PFOA and AOPs within AOP-wiki using epidemiological, *in vitro*, and *in vivo* published studies. Their results also reinforced the link between PFOA and PPAR-related pathways, which is consistent with the results of the pathway enrichment analysis we performed.

Recently, Chen et al. (2022) investigated the links between perfluorooctane sulfonate (PFOS) and molecular pathways using a similar approach and highlighted the response that this perfluorinated chemical induces to cholesterol- and fatty acid metabolism-related pathways (Chen et al., 2022). However, despite using a PBK model, the authors did not estimate the free concentrations of PFOS and only accounted for the extra quantity of albumin naturally produced and secreted to the media by the liver spheroids. Also, to derive their human equivalent doses they applied an uncertainty factor of 3 for toxicodynamic and 10 for toxicokinetic differences, which resulted in an overall UF of 30. Our use of a probabilistic PBK model accounted for the toxicokinetic UF. Therefore, the application of an UF of 3.16 to account for the toxicodynamic component was required in our study. The derivation of a similar HBGV to that of (Chen et al., 2022), where those authors did not estimate the free *in vitro* concentration of PFOS, may be explained by their use of an overall UF of 30. However, in both studies, the use of transcriptomics data coupled with PBK and QIVIVE allows for the identification of gene-pathway-level effects triggered by exposure to a given stressor, which can provide vital information to support chemical risk assessment. Another important point in common to both studies is the potential to identify different, and still comparable, sensitive pathways and diseases that would not be detected without the use of “omics data.”

In conclusion, we have developed an *in silico* workflow that integrates transcriptomics data with a calibrated PBK model and quantitative *in vitro* to *in vivo* extrapolation algorithm to derive a tolerable daily intake for perfluorooctanoic acid. Our approach incorporated an estimation of the *in vitro* free concentrations of PFOA and a pathway enrichment analysis that allowed us to identify the most sensitive molecular pathway related to the exposure to this “forever chemical”.

## Data Availability

Publicly available datasets were analyzed in this study. This data can be found here: https://www.ncbi.nlm.nih.gov/geo/query/acc.cgi?acc&equals;GSE144775.

## Footnotes

1
https://www.gnu.org/software/mcsim/ (19/01/2023).

2
https://github.com/GMPtk/MCSimViaRtools (19/01/2023).
