# Supplementary material for: A novel method to derive a human safety limit for PFOA by gene expression profiling and modelling
Source: Front Toxicol. 2024 Mar 21;6:1368320. doi: 10.3389/ftox.2024.1368320 (PMC10991825; doi:10.3389/ftox.2024.1368320)
Supplement: Supplementary file 2 [file DataSheet1.docx]

***Supplementary Material***

**A novel method to derive a human safety limit for PFOA by gene expression profiling and modelling**

Arthur de Carvalho e Silva^1,2*^, George Loizou^3^, Kevin McNally^3⸸^, Olivia Osborne^4^, Claire Potter^4^, David Gott^4^, John K. Colbourne^1,2^ and Mark R. Viant^1,2^

^1^ School of Biosciences, University of Birmingham, Birmingham, B15 2TT UK

^2^ Centre for Environmental Research and Justice (CERJ), University of Birmingham, Birmingham, B15 2TT UK

^3^Health and Safety Executive, Harpur Hill, Buxton, SK17 9JN UK

^4^Science Evidence and Research Division, Food Standards Agency, London, SW1H 9EX UK

^⸸^ Present address: Certara UK Limited, Simcyp Division, Sheffield, UK, S1 2BJ

*** Correspondence:**Arthur de Carvalho e Silva
a.csilva@bham.ac.uk

Keywords: PFOA, PBK, *in silico*, Bayesian, Markov Chain Monte Carlo, reverse dosimetry, omics, NAMs

**Supplementary File 2**

**Table S1.** Required parameters to configure the in vitro mass balance model for estimating freely dissolved concentrations of perfluorooctanoic acid.

| **MW (g/mol)** | **Melting point (°C)** | **pKa** | **Octanol-water partition ratio of the neutral form (log K_OW,N_)** | **Air-water partition ratio of neutral form (log K_AW,N_)** | **Water solubility (mg/L)** | **Cell type and assay conditions** |
| --- | --- | --- | --- | --- | --- | --- |
| 414.1 | 55 | 2.80 | 4.81 | -1.04 | 3300 | Human HepG2  384 well;  FBS = 10% |

**Table S2**. Number of differentially expressed genes identified and reported by Rowan-Carrol et al (2021) after exposing human liver spheroids to different concentrations of perfluorooctanoic acid over 14 days of exposure.

| **PFAS** | **Day** | **0.02 µM** | **0.1 µM** | **0.2 µM** | **1 µM** | **2 µM** | **10 µM** | **20 µM** | **50 µM** |
| --- | --- | --- | --- | --- | --- | --- | --- | --- | --- |
| PFOA | 1 | 0 | 8 | 36 | 8 | 19 | 79 | 69 | 227 |
|  | 4 | 22 | 30 | 35 | 41 | 14 | 42 | 100 | 184 |
|  | 10 | 14 | 7 | 2 | 4 | 10 | 82 | 101 | 593 |
|  | 14 | 4 | 0 | 8 | 9 | 6 | 76 | 96 | Cytotoxic |
